# Electrocardiographic Study in Adult Homozygous Sickle Cell Disease Patients in Lagos, Nigeria

**DOI:** 10.1155/2016/4214387

**Published:** 2016-09-21

**Authors:** Adedoyin Dosunmu, Akinsegun Akinbami, Ebele Uche, Adewumi Adediran, Sarah John-Olabode

**Affiliations:** Department of Haematology and Blood Transfusion, Faculty of Clinical Sciences, College of Medicine, University of Lagos, Lagos, Nigeria

## Abstract

*Background*. This study sought to identify the pattern of electrocardiographic changes in steady state adult sickle cell anaemia.* Methods.* A case-control, cross-sectional study was conducted amongst sickle cell patients attending the sickle cell clinic of Lagos State University Teaching Hospital, Ikeja, and HbAA controls. All consenting participants had haemoglobin electrophoresis done and were subjected to electrocardiography (ECG). The descriptive data were given as means ± standard deviation (SD). The differences were considered to be statistically significant when the* p* value obtained was <0.05.* Results.* A total of ninety-three sickle cell anaemia (SCA) patients and ninety haemoglobin AA (controls) were enrolled. There was no significant difference in the age of the participants with SCA and that of the controls but the body mass index was significantly higher in controls (*p* = 0.0001). Overall, 73.1% (68 of 93) had abnormal ECG while only 2 of 90 (2.2%) of controls had abnormal ECG. The common abnormalities observed were left ventricular hypertrophy, biventricular hypertrophy, and right ventricular hypertrophy.* Conclusion.* Patients with SCA in steady state tend to have normal heart rate but about 50% of them would have had ECG changes before the age of 20 years. ECG being a noninvasive test may be used to identify patients at risk for early intervention.

## 1. Introduction

Sickle cell anaemia is a genetic abnormality involving the haemoglobin. Patients present with a wide spectrum of disorders because of a single-point mutation in which thymine substitutes for adenine, thereby encoding valine instead of glutamine in the sixth position of the beta-chain.

The repeated sickling and unsickling damage the red cell membrane leading to irreversibly sickled red cell even when the oxygen pressure is increased. This causes reduced red cell life span as a result of membrane damage. Haemolysis consequent on the damaged red cell membrane could be intravascular or extravascular causing chronic anaemia.

Chronic anaemia is largely responsible for cardiac manifestations of sickle cell disease [1]. Sickle cell cardiomyopathy may also result from recurrent vasoocclusion with episodes of ischemia-reperfusion injury to multiple organ systems [2]. Progressive vasculopathic complications due to inflammatory and oxidative stress associated with sickling, intravascular haemolysis, and increased expression of cellular adhesion molecules contribute to progressive cardiac lesions [3]. Chronic anaemia causes increased cardiac output with minimal increase in heart rate [3]. Left ventricular stroke volume increases with significant dilatation of the left ventricle [4]. The dilated left ventricle adapts to the increased wall stress by developing eccentric hypertrophy in which wall thickening increased and myofibrils are elongated [5]. There is therefore increased left ventricular mass with age and left ventricular filling impairment [5–7]. Diastolic dysfunction by Doppler parameters is common in children and adults and it is an independent risk factor for mortality with a risk ratio of 4.8 [8]. The combination of diastolic dysfunction and pulmonary hypertension increased this risk to above 13 [5].

Electrocardiographic evidences of cardiomegaly and biventricular hypertrophy are common findings in sickle cell disease patients [9]. These are secondary to an increase in cardiac output in an effort to compensate for chronic anaemia that is seen in sickle cell anaemia [9]. There is a high output state and the resulting cardiomegaly increases the preload [10]. The increased preload and decreased afterload compensate for the left ventricular dysfunction and maintain normal ejection fraction and high cardiac output [11]. Other reported electrocardiographic abnormalities amongst the adult Nigerians are increased p-wave, QTc depression, and ST segment elevation [12, 13]. These show evidence of myocardial stress.

Skin fat and thin chest wall in addition to normal racial variation in black population may contribute to high voltages recorded in black sickle cell population [12, 13]. Hence caution should be taken in interpreting electrocardiogram in sickle cell patients.

Electrocardiographic change is common in sickle cell anaemia and it is associated with chronic anaemia. It is a noninvasive procedure; it is affordable in low-income countries where patients pay for every investigation and does not require extensive training. Presently, there is paucity of validated means to assess adult at risk of organ failure in steady state so that early intervention can be commenced as in the use of transcranial carotid artery Doppler scan in children. The prognostic value of electrocardiography is yet to be determined. This study therefore sought to determine pattern of electrocardiographic changes in adult patients in steady state attending the outpatient clinic.

## 2. Methods

A case-control, cross-sectional study was conducted amongst sickle cell patients attending the adult sickle cell clinic of Lagos State University Teaching Hospital, Ikeja, and HbAA controls between September and December 2014. Ethical approval was obtained from the institution's Ethics and Research Committee. Written and verbal consents were obtained from each participant. Participants were asked to fill structured questionnaires including demographic information, previous history of crises, date of last crisis, and cigarettes and alcohol intake. Inclusion criteria were patients with haemoglobin phenotype SS in steady state, no history of crises in the past 3 months established by a careful history, and complete physical examination. Exclusion criteria were haemoglobin phenotype SC patients, hypertensive patients, and HIV infected patients. Consenting participants consisting of medical students, nurses, administrative members of staff, and medical doctors with haemoglobin phenotype AA were also enrolled as controls. All consenting participants had haemoglobin phenotype confirmed, subjected to electrocardiography (ECG), and the females were nonpregnant.

### 2.1. ECG Procedure

Age, weight, height, BMI, sex, and medications of all patients were recorded. Ten leads were placed at precise anatomical locations to obtain quality data. The four limb lead electrodes were applied to the extremities starting with the right leg and then the left leg, right arm, and left arm. All the chest leads were also applied at the precordial electrode locations (V1–V6). ECG was recorded after the connection of the limb and chest leads. ECG tracings were read and interpreted by a cardiologist. Randomly selected ECG tracing readings were double checked by an independent cardiologist. Measurements of heart rates, QTc, and PR intervals were done in the standard fashion [13]. ECG reference values for Nigerian population were used as cutoff values for duration of electrocardiographic deflections and intervals [13]. Left ventricular hypertrophy diagnosis was based on Sokolow and Lyon voltage criteria [14]. Right ventricular hypertrophy diagnosis was based on the Allenstein and Mori criteria [15]. Left atrial dilatation diagnosis was based on the criteria described by Marcus and validated in the Negro population by Araoye [16]. ECG model was 11D by Dong Jiang, manufactured by CMICS Medical Instruments Co. Ltd., China.

## 3. Analysis of Data

The descriptive data was given as means ± standard deviation (SD). The Pearson correlation was used for the analytical assessment. To assess association between two discrete parameters the Fisher Exact Test was used to determine the odds ratio. The differences were considered to be statistically significant when the* p* value obtained was <0.05.

## 4. Results

A total of ninety-three sickle cell anaemia (SCA) patients and ninety haemoglobin AA (controls) were enrolled. The proportion of females was 54% amongst SCA patients and 50% amongst controls (Table 1). The mean age was of 24.55 ± 9 years with a median of 22 years in SCA while the mean age in controls was 24 ± 7 years and median age was 23 years. There was no significant difference in the age. The mean BMI in SCA was 19 ± 3.3 kg/m^2^ with a median of 18.9 kg/m^2^ while the mean BMI in controls was 22.5 ± 3.1 kg/m^2^ and the median was 21.2 kg/m^2^. The difference was highly significant (*p* = 0.0001). Spearman correlation analysis of age and BMI showed positive correlation (*r* = 0.23 and *p* = 0.03).

The following electrocardiographic abnormalities were observed in SCD participants: left ventricular hypertrophy (43%), biventricular hypertrophy plus right ventricular hypertrophy (28%), right atrial dilatation (1.16), premature ventricular complex (0.01%), 1st-degree atrioventricular block (0.06%), and myocardial strain (0.01%) (Table 2). Left ventricular hypertrophy is the most common one and it occurs in 20% of adolescents. Amongst adolescents, 50% have one abnormality or another (Table 3).

Overall, 73.1% (68 of 93) had abnormal ECG while only 2 of 90 (2.2%) of controls had abnormal ECG. The percentage of SCA males with abnormal ECG was 88% while 60% of females had abnormal ECG. A total of 11 of 42 (26.1%) males with SCA had left ventricular hypertrophy and 7 of 42 (16.6%) males with SCA had biventricular hypertrophy and right ventricular hypertrophy. A total of 9 of 51 (17.6%) females with SCA had left ventricular hypertrophy while 7 of 51 (13.7%) had biventricular hypertrophy and only 2 of 51 (3.9%) had right ventricular hypertrophy.

Despite the similarity in mean heart rate of SCA patients and controls, 77.28 ± 14.61 and 77.19 ± 15.30 seconds, respectively, this was not statistically significant (*p* = 0.847). The mean QTc interval of SCA patients was 0.38 ± 0.035 seconds and controls 0.37 ± 0.02 seconds (*p* = 0.123). The mean PR interval of SCA was 0.186 ± 0.06 seconds and controls 0.169 ± 0.036 seconds (*p* = 0.369). The mean QRS duration of SCA was 0.07 ± 0.09 and controls 0.043 ± 0.14 seconds (*p* = 0.055).

Correlation analysis of changes in age and BMI with ECG intervals showed significant correlation only between age and the PR interval (*r* = 0.3; *p* = 0.007), Table 4.

There was no significant association between the presence of LVH and frequency of bone pain crisis using Fisher's Exact Test (odds ratio = 0.85, *p* = 0.79), Table 5. Similarly, there was no significant difference between the PR interval of those with frequent bone pain crisis and those with painful crisis below 3 times in one year (*p* = 0.43).

## 5. Discussion

This study demonstrated electrocardiographic abnormalities disparity between the sickle cell anaemia and the controls participants, in keeping with previous researchers [12, 13, 17]. This study reported that only 2.2% of the age-matched controls had electrocardiographic abnormalities compared with 73.1% of sickle cell disease patients. This is similar to that of Holloman et al. [17], who reported that 72% sickle cell anaemia participants had electrocardiographic abnormalities. The 2.2% prevalence amongst controls that had abnormal ECG in this study is also similar to 3.2% of controls reported by Oguanobi et al. [13].

The common cardiac abnormalities amongst sickle cell anaemia patients may contribute to increased morbidity, mortality, and sudden death amongst them. Many factors are responsible for the observed increase in cardiac abnormalities in sickle cell disease; the most important one of them is the chronic anaemia which is a sine qua non of sickle cell disease [18, 19]; other factors are increased pulmonary vascular disease, peripheral vascular disease, myopathy [20], and increased myocardial iron deposition as demonstrated by autopsy studies [21]. However, increased myocardial iron deposition was not supported by magnetic resonance imaging studies using T2^*∗*^ measurements which could not demonstrate increased myocardial iron deposition even in the presence of a significant transfusion history, systemic iron overload, and/or hepatic iron overload [22].

The most frequent electrocardiographic abnormality amongst SCA participants reported in this study and almost all previous works was left ventricular hypertrophy [12, 18, 23]. A total of 43% had left ventricular hypertrophy (LVH) and this was found to be more frequent in males than females. This is similar to 48% obtained by Aluko [23]. Lindsay Jr. et al. [18] also reported that LVH was more frequent in males than females but LVH was only seen in 22% of SCA patients studied. This is much lower than 75% of participants reported by Oguanobi et al. though they reported a higher percentage (96.7%) of ECG abnormalities amongst SCA patients [13]. The higher mean age of participants reported by Oguanobi et al. compared with the present study could account for a higher prevalence of LVH in their study. Increasing age was reported to be directly proportional to increasing LV filling [5].

Left ventricular stroke volume increases with significant dilatation of the LV as a result of chronic anaemia and a subsequent increase in cardiac output [24]. The degree of LV dilatation is directly proportional to the degree of anaemia [25].

Diastolic dysfunction, an independent mortality risk factor, is common in SCA children [8]. In this study, LVH was observed in 20% of adolescents. Moreover, it was observed to be independent of painful crisis. Left ventricular hypertrophy appears therefore to be an independent prognostic feature that appears early in life.

In this study, ECG evidence of biventricular hypertrophy was next to left ventricular hypertrophy in sickle cell anaemia. This was present in a total of 30.3% of participants. However, sickle cell disease patients in steady state without pulmonary hypertension were reported to have dilated right heart chambers without significant right ventricular dysfunction [8]. Acute pressure overload in the presence of chronic pulmonary vasculopathy is felt to be the reason for acute right ventricular decompensation and pulmonary hypertension.

The mean heart rate of SCA was significantly different from that of controls (*p* = 0.847) despite similarity in values. Chronic anaemia associated with sickle cell anaemia minimally increases heart rate despite a marked increase in cardiac output [3]. This reflects adequate cardiac compensation in steady state sickle cell anaemia.

The QTc and PR intervals were not significantly different in SCA and controls. However, the QRS duration was somewhat significantly higher in SCA than in controls in this study (*p* = 0.055). This is at variance with findings by others, where ECG intervals in SCA were significantly higher than in controls. This may be due to the exclusion criteria that excluded the patients that had crisis within 3 months of study. Patients that were doing well must have been selected. This suggests that even amongst SCA patients with low frequency of crisis there are ECG abnormalities. The presence of left ventricular hypertrophy may therefore be used to identify patients at risk for early intervention.

In conclusion, cardiac abnormalities particularly pulmonary hypertension and diastolic left ventricular dysfunction have been known to be risk factors of syncope and sudden death in SCA [3]. ECG being a simple tool may be used to assess cardiac abnormalities for early intervention. The chronic anaemia of sickle cell anaemia is usually adequately compensated for by increased cardiac output among other compensatory mechanisms as shown by the normal heart rate. However, degree of anaemia correlates significantly with increase in left ventricular muscle mass secondary to compensatory hypertrophy that occurs in response to vascular dilatation [26]. Therefore, the ECG may be a simple and noninvasive tool for assessing prognosis in the adult sickle cell clinic since these changes occur early in adolescents in steady state. It is therefore suggested that ECG should be done annually for SCA patients and those with LVH should be placed on hydroxyurea as primary prophylaxis. This study should stimulate the use of ECG to assess adult SCA patients for early intervention to prevent cardiac events particularly in low-income countries where cost of more reliable investigations may be an issue.

## Figures and Tables

**Table 1:** The demographic data.

Parameter	Sickle cell disease	Controls	*p* value
Number	93	90	
Male : female	42 : 51	45 : 45	
Mean age (years)	24.55 ± 9.01	24 ± 7.01	0.69
Mean BMI	19.21 ± 2.78	22 ± 3.1	0.0001
Mean age for males (years)	24.12 ± 9.37	25.43 ± 4.78	0.42
Mean age for females (years)	24.92 ± 8.77	22.56 ± 3.98	0.065
Rate	77.28 ± 14.6	77.19 ± 15.3	0.84

**Table 2:** The overall ECG abnormalities of SCD.

Parameters (number of participants: 93)	Present
Left ventricular hypertrophy	40 (43%)
Right ventricular hypertrophy	26 (28%)
Left atrial enlargement	1 (1.16%)
Right atrial enlargement	0 (0%)

**Table 3:** ECG abnormalities by age in SCD.

Age range (years)	LVH	BVH	RVH
<20	20%	16%	14%
21–30	28%	18%	14%
>30	23%	14%	5%

Key: LVH—left ventricular hypertrophy; BVH—biventricular hypertrophy; RVH—right ventricular hypertrophy.

**Table 4:** Correlation analysis.

	Age	BMI
PR	*r* = 0.3; *p* = 0.007	*r* = 0.075; *p* = 0.57
QRS	*r* = 0.22; *p* = 0.25	*r* = −0.25; *p* = 0.2
QTc	*r* = 0.16; *p* = 0.67	*r* = 0.045; *p* = 0.5
Heart rate	*r* = 0.08; *p* = 0.45	*r* = 0.091; *p* = 0.39

**Table 5:** LVH and frequency of crises.

	>3 crises/yr	<3 crises/yr	Total
LVH present	8 (9%)	33 (38%)	41 (48%)
LVH absent	10 (12%)	35 (41%)	45 (52%)

Total	18 (21%)	68 (79%)	86 (100%)

Key: LVH—left ventricular hypertrophy; /yr—per year.
